# Understanding the responsiveness of nitric oxide to acute eccentric resistance exercise in elderly obese women

**Published:** 2016-06-19

**Authors:** Tatiane Gomes Teixeira, Dahan da Cunha Nascimento, Ramires Alsamir Tibana, Nuno Manuel Frade de Sousa, Vinicius Carolino de Souza, Jeeser Alves de Almeida, Amilton Vieira, Octavio Luiz Franco, Guilherme Borges Pereira, Jonato Prestes

**Affiliations:** 1 *Catholic University of Brasilia, Graduation Program on Physical Education, Brasilia, Brazil*; 2 *Laboratory of Exercise Physiology, Faculty Estacio of Vitoria, ES, Brazil*; 3 *Graduation Program in Gerontology, Catholic University of Brasilia, Brasilia, Brazil*; 4 *Physical Education Department, Federal University of Mato Grosso of South, Brazil*; 5 *University of Brasilia, Brasilia, Brazil*; 6 *Graduation Program on Biotechnology and Genomic Sciences, Catholic University of Brasilia, Brazil*

**Keywords:** aging exercise, nitric oxide synthase, nitrite, obesity, responders

## Abstract

**Background and Aim:**

The aim of the present study was to examine nitrite concentration responses following eccentric resistance exercise (ERE) in elderly obese women. We also investigated the existence of high (HR) and low responders (LR) for nitrite and the possible differences with respect to creatine kinase (CK) response, metabolic and body composition variables.

**Methods:**

Forty-nine elderly obese women completed an ERE session utilizing knee extensor exercise. LR for serum nitrite were defined as a ∆nitrite ≤ 20th percentile or 24.1 µmol/L and HR as a ∆nitrite > 20th percentile.

**Results:**

Ten subjects were classified as LR and the remaining as HR (n = 39). The HR group displayed greater nitrite concentration at 0 h, 3 h, 24 h and 48 h following the ERE as compared with the LR (*p* < 0.05), and CK increased after 24 h and 48 h only for the HR group following the ERE (*p* < 0.05). Peak nitrite concentration was higher in the HR group versus the LR group (*p* < 0.05), while there was no difference between groups for pre-exercise nitrite values. The LR group displayed higher (*p* < 0.05) body fat, cholesterol, LDL and lower upper limb fat-free mass as compared with the HR group. The LR had lower (*p* < 0.05) upper limb fat-free mass than the HR group.

**Conclusions:**

Elderly obese women classified as HR displayed higher nitrite responses to ERE. Thus, researchers should be aware of the presence of different responsiveness of nitrite to acute exercise to avoid misinterpretation of data and to identify the higher cardiovascular risk factor of those classified as LR.

**Relevance for patients:**

The elevated NO up to 48 h following an ERE session may suggest an important protective cardiovascular effect. The higher body fat, cholesterol, LDL and lower upper limb fat-free mass in the LR group might represent a deleterious effect of lower serum levels of nitrite.

## 1. Introduction

The population of elderly individuals has increased worldwide and women live an average of seven years longer than men [1]. Increasing age compromises functional capacity to perform activities of daily living accompanied by increased prevalence of overweight, obesity, metabolic syndrome, augmented cardiovascular risk factors, as well as loss of muscle mass and strength (e.g. sarcopenia) [2]. Among biological changes with advancing age, lower nitric oxide (NO) bioavailability has been suggested as an important risk variable, affecting many tissues, including cerebral, cardiac, bone and musculoskeletal organs [3].

NO exerts important physiological roles, including regulating blood pressure, acting on vascular tone, inhibiting monocytes/macrophages adhesion (anti-atherosclerotic), anti-thrombotic by inhibiting platelet aggregation and anti- inflammatory effects by inhibiting nuclear transcription factor (NFkB) [4]. Thus, dysregulation of NO concentrations has been recognized as an important risk factor in metabolic and cardiovascular diseases [5]. Regular exercise exerts positive effects on NO bioavailability and endothelial function in the elderly [6], suggesting a positive influence of a physically active lifestyle on diseases such as atherosclerosis and hypertension [7,8]. Additionally, significant NO increases following a second effort exercise test (24 h after the first) exerted a protective cardiovascular effect (ST depression was lower for the second test) in subjects aged between 41 and 74 years (mean 56.2 ± 1.6 years) with ischemic heart disease [9].

Although physical exercise may provide beneficial effects on health in elderly, some subjects may not respond appropriately, or may even show adverse metabolic responses [10]. Interestingly, elderly subjects preserve eccentric strength as compared with concentric strength, while an eccentric protocol induces lower perceived exertion and metabolic stress versus conventional resistance exercise [11], and induces no alarming creatine kinase (CK) responses in obese elderly women [11]. Changes in serum CK concentration has been previously used as an indicator of muscular damage [12], and is still used in recent studies [13]. A particular problem in using serum CK concentration is due to its inter-subject variability, especially following exercise [14]. Similar baseline serum CK values and notable inter-subject variability after exercise, justifies classification of low and high responders and it can be partially explained by body composition [15], training status [15], and genetic factors [16].

Eccentric resistance exercise (ERE) also induces an acute decrement of pro-inflammatory markers, possibly indicating a transient protection against the low-grade inflammation present in obese elderly women [17]. ERE also increases vascular endothelial growth factor and endothelial nitric oxide synthase (eNOS) gene expression [18].

Chronic risk factors for cardiovascular disease such as high body fat percentage and hyperlipidemia impair basal nitricoxide-mediated dilatation [19]. A high pro-inflammatory status influenced by a high body fat percentage might impair muscle strength, negatively affecting fat-free mass in obese elderly women [20], decreasing endothelium-dependent relaxation and inhibiting agonist-stimulated production of NO. Consequently, baseline metabolic and body composition variances between HR and LR subjects might affect NO response following an ERE session. It is important to note that in the present study, serum nitrite concentration was measured in order to reflect the NO concentration.

Thus, the aim of the present study was to examine nitric oxide concentration responses following ERE in elderly obese women, and to assess the existence of high responders (HR) and low responders (LR) for nitric oxide. The second aim was to verify if the LR group would present higher metabolic disturbances at baseline when compared with HR participants and to compare the acute CK response between groups. Our hypothesis was that a large range of nitrite response to ERE would be observed among participants, making it possible to identify different levels of responsiveness. Another hypothesis was that LR participants would present higher metabolic disturbances at baseline as compared with HR participants.

## 2. Materials and Methods

### 2.1. Participants

Forty nine elderly women with an average age of 69.7 (67.8–71.5, CI 95%) years, average body weight of 76.0 kg (72.4.7–79.6), average height of 151.3 cm (149.7–152.9), and average body mass index (BMI) of 33.1 kg/m^2^ (31.5–34.8) from a local community located in the Federal District, Brazil were invited to participate in the present study (see Table 1 for participants’ characteristics). Inclusion criteria were as follows: age ≥ 60 years, sedentary females, body fat percentage > 30% and completion of all anthropometric testing. Obesity was determined according to the recommendations of the National Institute of Diabetes and Digestive and Kidney Diseases, assuming a cut-off point of 30% for women. Individuals were considered sedentary according to their responses on the International Physical Activity Questionnaire. Participants with inflammatory, rheumatic, or autoimmune conditions or use of medications (i.e., beta blockers, hormone replacement therapy, anti-inflammatory, insulin) or any type of disease that could compromise the tests and procedures were excluded. The study was approved by the Institutional Research Ethics Committee in accordance with the Declaration of Helsinki, and all participants gave written informed consent before inclusion in the study.

### 2.2. Criteria for the determination of nitrite responsiveness

Low responders for serum nitrite were defined as a ∆ nitrite ≤ 20th percentile or 24.1 µmol/L and high responders as a ∆ nitrite > 20th percentile. Due to the lack of data with serum nitrite response to resistance exercise, we defined low responders according to the definition of a “rare event” as compared with high responders and no clinical or physiological criteria have been established to define the serum nitrite level at which an individual would be considered a HR [15]. Moreover, the value of 24 µmol/L was an increase of approximately 50% in serum nitrite concentration following ERE, and most participants had an increase of more than 50% in serum nitrite concentration. According to this *a priori* definition, we assumed that the ∆ nitrite ≤ 20th percentile or 24.1 µmol/L was a valid criterion to define LR and HR for this marker.

**Table 1.** Mean (95% confidence interval) of anthropometric, biochemical, leg extension 10 RM and inflammatory parameters for low (LR) and high (HR) responders groups.

	LR	HR	*p* value
Age, yr	69.5 (64.7 – 74.3)	69.7 (67.6 – 71.8)	0.917
Weight, kg	78.3 (69.7 – 86.9)	75.4 (71.2 – 79.5)	0.519
SBP, mmHg	125.7 (120.7 – 130.8)	122.9 (119.9 – 126.0)	0.376
DBP, mmHg	75.9 (73.4 – 78.4)	72.5 (70.8 – 74.3)	0.070
MBP, mmHg	92.5 (90.2 – 94.8)	89.3 (87.5 – 91.2)	0.115
Body fat, %	45.6 (40.3 – 50.8)	40.6 (39.0 – 42.3)	0.017
Body fat, kg	32.7 (22.6 – 42.8)	25.1 (22.8 – 27.3)	0.019
Fat-free mass, kg	36.5 (32.9 – 40.2)	36.7 (35.0 – 38.5)	0.933
UL fat-free mass, kg	3.5 (3.0 – 3.9)	3.9 (3.7 – 4.1)	0.045
LL fat-free mass, kg	11.7 (10.5 – 12.9)	11.1 (10.6 – 11.6)	0.182
BMI, kg/m2	34.3 (30.0 – 38.6)	32.8 (31.0 – 34.7)	0.489
Cholesterol, mg/dL	232 (203 – 260)	206 (195 – 217)	0.043
Triglycerides, mg/dL	143 (96 – 189)	135 (112 – 158)	0.747
HDL, mg/dL	48 (42 – 54)	47 (43 – 51)	0.835
LDL, mg/dL	155 (129 – 182)	132 (122 – 141)	0.044
Glucose, mg/dL	94 (89 – 99)	95 (88 – 102)	0.866
C-reative protein, mg/dL	0.19 (0.11 – 0.26)	0.33 (0.21 – 0.46)	0.643
Interleukin-6, pg/mL	5.36 (0.09 – 10.66)	4.97 (2.01 – 7.92)	0.445
Leg extension 10 RM, kg	35.2 (28.1 – 42.2)	34.3 (31.3 – 37.3)	0.786

BMI, body mass index; SBP, systolic blood pressure; DBP, diastolic blood pressure; MBP, mean blood pressure; UL, upper limb; LL, lower limb; HDL, high-density lipoprotein; LDL, low-density lipoprotein; RM, repetition maximum.

### 2.3. Nitrite and creatine kinase (CK) measures

Elderly women arrived at the laboratory between 8:00 and 10:00, after an overnight fasting. Blood samples of 5 mL were drawn from the antecubital vein into vacutainer tubes (Becton Dickinson, Brazil) pre- and 3, 24, 48 h post-exercise to analyze nitrite and CK kinetics. Samples were then centrifuged at room temperature at 2,500 rpm for 15 min. All participants were requested to avoid smoking, alcohol and caffeine consumption as well as unusual physical activity to avoid influence on these parameters. Serum was stored and frozen at −80°C for subsequent analysis. The serum concentration of nitrite was determined using the *Griess* reaction according to the descriptions by Zahedi Asl et al. [5]. Serum samples were deproteinized by adding zinc sulfate (15 mg/mL) and were centrifuged at 10000 g for 10 min; 100 μL of the supernatant was added to a microplate well, and 100 μL of vanadium (III) chloride (8 mg/mL), Griess reagents (50 μL sulfanilamide (2%) and 50 μL N-(1-naphthyl) ethylendiamine dihydrochloride (0.1%)) were added. After 30 min of incubation at 37°C, absorbance was read at 540 nm using a microtiter plate reader (Sunrise, Tecan, Austria). Nitrite concentration was determined from the linear standard curve established by 0-100 µmol/L sodium nitrite. CK concentration was determined by use of a commercially available Reflotron CK assay using the Reflotron system (Boehringer-Mannheim, Mannheim, Germany).

### 2.4. Biochemical parameters

The lipid profile (GPO/POD) was measured using the enzymatic colorimetric method on Autohumalyzer equipment (Boehringer-Mannheim, Mannheim, Germany) at baseline only. No measures were provided immediately after 3, 24 and 48 h post-exercise. High density lipoprotein-cholesterol (HDL- C) HDL-C was determined by ionic exchange followed by colorimetric reaction with the Linco® Research Inc. kit (St Louis, USA), and blood glucose was measured by hexokinase enzymatic assay. Interleukin-6 (IL-6) concentration was measured by Quantikine high sensitivity commercial enzyme-linked immunosorbent assay Kit (R&D Systems, Minneapolis, MN, USA). The intra-assay coefficient of variation of the kits was 1.2%-4.3% and the interassay coefficient of variation was 5.3%-6.7% for IL-6. Plasma C reactive protein (CRP) was determined using high-sensitivity turbidimetry according to the manufacturers’ protocol (Boehringer-Mannheim, Mannheim, Germany). The intra assay coefficient of variation of the kits was 1.7%-5.8% while the inter assay coefficient of variation was 3.3%-5.4%. The measurements of nitrite, CK, IL-6 and CRP were performed in triplicate and averaged. The intra-assay coefficient of variation for nitrite and CK was 2.1%-3.5% and 4.6%-6.1% while the inter assay coefficient of variation was 2.3%-4.3% and 3.6%-5.7%, respectively.

### 2.5. Ten repetition maximum test

The ten repetition maximum (10RM) test was used to determine each individual’s load to be used during the ERE session. We adopted the protocol used in a previous study from our research group [18]. The elderly women visited the laboratory on two occasions. On the first visit they completed a recall form and physical activity questionnaire, anthropometric measures, dual-energy X-ray absorptiometry (DXA) analysis and completed an adaptation session on a leg extension isoinertial machine (Righetto, Sao Paulo, SP, Brazil) utilizing three sets of 8–10 submaximal repetitions. Three days later they performed the 10RM test. Participants rested three days and completed the 10RM test again to determine test-retest reproducibility (R = 0.98). The test was terminated at the moment participants were unable to perform the complete movement or when voluntary concentric failure occurred. The following strategies were adopted to reduce test variations: a) standard instructions were given before the test so that the volunteer was aware of the entire routine to be performed during data collection; b) the subject was provided technical instruction on the exercise performance; c) the evaluator was aware of the position adopted by the practitioner during the test, since slight variations in the positioning of the joints involved in the movement could trigger other muscles, leading to misinterpretation of the obtained scores; d) the participants were verbally encouraged with the purpose to keep their motivation level high; e) the additional load used in the study was previously measured on a precision scale. Rest intervals between trials in each exercise during the 10RM test were set between 3 and 5 min. Relative muscle strength was calculated by means of the following formula: relative strength = absolute strength (kg)/body mass (kg). The participants refrained from any stimulating substance (caffeine or alcohol) and did not perform physical activity during the week prior to experimental testing. The 10 RM tests were scheduled between 14:00 and 16:00 and were performed under standardized controlled room temperature. The knee extension exercise was chosen because lower limb strength in the elderly is particularly important, as it is strongly affected by sarcopenia and loss of functionality [2]. Moreover, the choice for lower body ERE using leg extension exercise was based on the greater decline of muscle strength as compared with the upper limbs [21].

### 2.6. Acute eccentric resistance exercise (ERE)

Seven days after the adaptation session and 10RM tests, participants completed an acute ERE [22]. The session started with a series of general warming of the lower extremities on a cycle ergometer for 10 min at 60 rpm and 50 W, followed by a specific warm-up of 10 repetitions at 50% of 10RM, with a rest interval of 3 to 5 min. The ERE session was performed on the bilateral knee extension isoinertial machine with a load corresponding to 110% of the 10 RM. The repetition cadence was controlled so that the repetition of the eccentric phase was performed in 3 s, with the concentric phase moved by the test administrators. Individuals completed seven sets of 10 repetitions with a passive rest of 1 min between sets. The range of motion was limited between 0 and 90°.

### 2.7. Body composition

Body fat percentage and lean body mass were determined by dual-energy X-ray absorptiometry (General Electric-GE model 8548 BX1L, year 2005, Lunar DPX type, software Encore 2005; Sarstedt, Rommelsdorf, Germany). The tests included a complete body scan of the volunteers, in the supine position, lasting approximately 17 minutes, with the apparatus always calibrated and operated by a technically trained professional.

### 2.8. Statistical analysis

The results were expressed as means (95% confidence interval). Shapiro-Wilk tests were applied to check for the normality distribution of the study variables. In case of nonparametric distribution, a logarithmic transformation was performed to approximate a normal distribution. The difference between baseline nitrite and peak nitrite concentration (the highest value achieved at 0, 3, 24, or 48 h for each subject), or ∆ nitrite, was considered the response following exposure to the ERE. The achieved power of the sample size was determined using G*Power version 3.1.5 (Kiel, Germany), based on the differences between baseline and peak concentrations of nitrite between the HR and LR groups. For nitrite sample size (n = 49), the effect size *d* was large and the power was 0.99. Strength, anthropometric and blood parameter comparisons between the LR and HR groups were determined by independent t test. A mixed model ANOVA was used to compare the differences in nitrite and CK concentration between nitrite responder groups at pre-exercise and over the course of 48 h post-exercise. Compound sphericity was verified by the Mauchley test. When the assumption of sphericity was not met, the significance of F-ratios was adjusted according to the Greenhouse–Geisser procedure. Simple main effects were used to determine the difference between groups at each time point and to determine the difference between time points within each group. The level of significance was *p* ≤ 0.05 and SPSS version 20.0 (Somers, NY, USA) software was used.

## 3. Results

**Figure 1.**
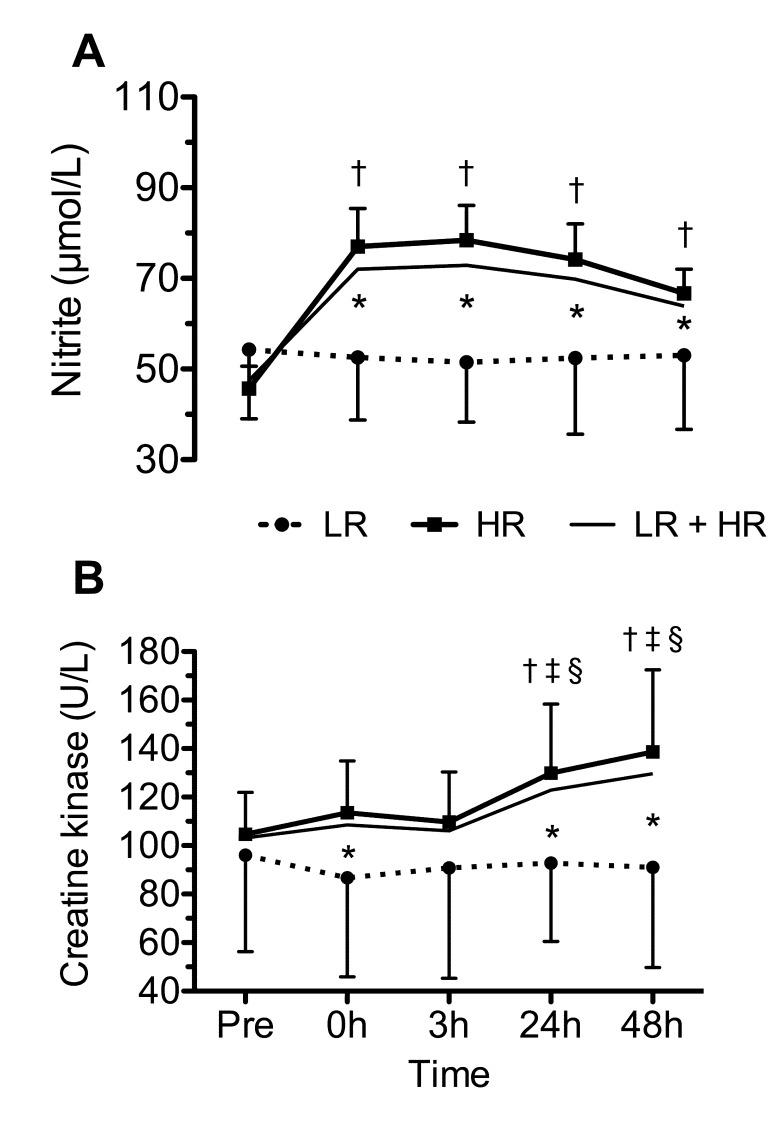
Means (95% confidence interval) of serum nitrite (A) and creatine kinase (B) in low (LR),high (HR) responder groups and LR + HR group pre and at 0 h, 3 h, 24 h and 48 h following the eccentric resistance exercise session. *Significantly different versus the LR group; † Significantly different versus pre for HR group; ‡ Significantly different versus 0 h for HR group; § Significantly different versus 3 h for HR group (*p* ≤ 0.05).

Among 49 participants, 10 (20.4%) were classified as LR according to our pre-determined statistical criteria for increases in serum nitrite concentration. The mean increase in serum nitrite concentration (∆ nitrite) for the LR, 12.2 (95% CI: 4.5 – 19.9) µmol/L, was significantly lower (*p* < 0.001) than the HR group, 48.7 (95% CI: 42.2 – 55.3) µmol/L. The time-course of serum nitrite among all participants and in the LR and HR groups is shown in Figure 1A. Considering that the LR group was defined as a ∆ nitrite ≤ 20th percentile, it is expected that the response of the HR group represents the regular response, as confirmed by the response of LR + HR groups (Figure 1A). There was a statistically significant interaction between the LR and HR groups and time on serum nitrite concentration, *F*(2.876, 135.189) = 5.615, *p* = 0.001. The nitrite concentration increased immediately after and during the 48 h of the ERE for the HR group, while no significant differences were noted for the LR group across time points. No significant differences between groups were noted in the baseline serum nitrite concentration (LR, 54.4 (95% CI: 39.0 – 69.7) µmol/L and HR, 45.7 (95% CI: 40.8 – 50.6) µmol/L; *p* = 0.14). However, significantly greater serum nitrite concentration was noted for the HR group at 0, 3, 24 and 48 h following the ERE (*p* < 0.05). The peak nitrite concentration that occurs mainly between 0 and 3h after ERE for each subject, was significantly greater in the HR group versus the LR group (HR, 94.4 (95% CI: 87.6 – 101.2) µmol/L and LR, 66.5 (95% CI: 51.1 – 82.0) µmol/L; *p* = 0.001).

Figure 1B shows the time-course of serum CK concentration in the LR and HR groups for nitrite. Although there was no statistically significant interaction between the groups and time on CK concentration, *F*(3.187, 143.399) = 2.075, *p* = 0.086, the group response to the ERE was similar to the response of nitrite. The CK concentration increased over the time following ERE for the HR group, while no significant differences were noted for the LR group across time points. No significant differences between groups were noted in the baseline serum CK concentration LR, 96.0 (95% CI: 56.3–135.7 U/L) and HR, 104.7 (95% CI: 87.5–122.0 U/L; *p* = 0.64). However, significantly greater serum CK concentration was noted for the HR group at 0, 24, and 48 h following the exercise (*p* < 0.05).

Anthropometric, biochemical, leg extension 10 RM and inflammatory parameters mean values for the HR and LR groups, based on nitrite responsiveness, are shown in Table I. The LR group showed higher (*p* < 0.05) body fat, cholesterol and LDL than the HR group. The LR had lower (*p* < 0.05) upper limb fat-free mass than the HR group. All other anthropometric, biochemical variables and leg extension 10 RM were not significantly different between groups based on nitrite responsiveness. Lastly, the inflammatory markers (CRP and IL-6) were also not significantly different between LR and HR groups.

## 4. Discussion

The main finding of the present study was the existence of an HR group for nitrite in elderly obese women following an acute ERE session, while nitrite baseline values were the same for both groups. Elderly women classified as LR presented higher total cholesterol, LDL, body fat and lower upper limb fat-free mass as compared with the HR group, which might affect the response of nitrite following an acute ERE session. The classification for the HR group in the present study was a ∆ nitrite > 20th percentile or > 24.1 µmol/L. Among the 49 participants, 10 (20.4%) were classified as LR according to our pre-determined statistical criteria for increases in serum nitrite concentration. The HR group displayed greater serum nitrite concentration at 0, 3, 24, and 48 h following the ERE as compared with the LR. Additionally, only the HR group increased CK at 24 and 48 h following the acute ERE session. The peak nitrite was greater in the HR group versus the LR group, thus confirming the initial hypothesis.

In this sense, considering that nitrite measures directly reflect NO measures, the elevation of NO following an acute exercise session might represent a protective effect. Providing evidence toward this end, patients with ischemic heart disease aged 41 to 74 years completed two maximal symptom limited exercise tests (ETs) on a cycle ergometer with 24 h intervals (ET1 and ET2), which consisted of 25 W increments every 2.5 min. There was an increase of plasma NO only after ET2 along with a decrease of electrocardiographic consequences of a similar ischemic stress. The considered protective values were 23.6 ± 2.2 µmol/L post exercise test (during the recovery phase until 9 min or until angina and electrocardiogram changes disappeared) [23]. The nitrite levels of the present study remained elevated for 48 h in the HR group. However, different methods and techniques between the studies were utilized.

Furthermore, 23 women completed 20 minutes of a constant load exercise test at 90% of the anaerobic threshold (AT) and a maximal incremental test (IT). Results indicated a post-exercise hypotension following aerobic sessions in elderly women with hypertension accompanied by an elevation of salivary NO, reinforcing the role of this molecule on lowering exercise-induced blood pressure [24]. The results of the present study revealed that NO remained elevated up to 48 h following an ERE session, different from the immediate post exercise response found in a previous study [24]. This may suggest an important protective cardiovascular effect.

Furthermore, over weighted and obese women were submitted to exercise on a cycle ergometer with increasing loads every 3 minutes (50, 100 and 150 W) not exceeding 9 minutes [25]. NO increased immediately after exercise by 26.4 and 42.5% in the obese and controls respectively, which provides evidence that obesity compromises exercise-induced NO release. In the present study, NO increased more than 50% in the HR subjects, while in the LR group the elevation was < 50%. Although the HR group was obese, a beneficial NO increase was observed up to 48 h following an acute ERE session. Moreover, the LR group presented higher adiposity, total cholesterol and LDL, highlighting the existence of a possible body fat threshold that compromise exercise-induced NO release in elderly obese women or even unknown mechanisms that attenuate NO response to exercise.

Similarly, the existence of HR and normal responders (NR) groups has been identified for IL-6 and CK in elderly obese women who completed an acute ERE protocol [26]. Although ERE is suggested to induce greater muscle damage [27], the higher values of CK increase in this study were in average of 140 U/L for the HR group. To reinforce the safety of this method and considering that muscle is the most important source of CK release, greater levels are frequently associated with damage in this tissue. The adopted protocol for ERE induced no alarming CK responses, which could be indicative of excessive muscle damage. Furthermore, the CK reference values for women are 40 to 150 U/l [14). Although the magnitude of CK activity after exercise was small, when accompanied with NO increments after exercise in the HR subjects, it could be indicative of muscle damage and regeneration [28]. The greater NO response following an ERE session and the greater CK response in the HR subjects might infer a better adaptive response to injury and regeneration in these individuals. There is evidence that some NO release is necessary for an efficient muscle repair [29]. In this sense, the elevation of NO following an acute ERE session in the HR group accompanied with greater CK response when compared with LR subjects might represent a protective effect.

A recent study was designed to investigate the NO kinetic response following an ERE session with the same protocol adopted in this study and the possible effect of Glu298Asp eNOS gene polymorphism in 87 elderly women [30]. The results of the study demonstrated that NO remained elevated for up to 48 h following an acute ERE session as compared to baseline for GG (49.0 µmol/L) and GT/TT (46.5 µmol/L) groups, without genotype interaction, while no analyses of responsiveness was conducted. The mean increase in serum nitrite concentrations for the LR participants (12.2 µmol/L) was significantly lower than the HR group (48.7 µmol/L) in this study. This reinforces the limitation in the literature of reporting only the group mean and standard deviation for exercise or training responses [31]. Furthermore, the variation in response around the mean alludes to the inter-individual variation in exercise and training responses. This confirms the importance of studying individual responsiveness to exercise.

Although the data in this study demonstrates interesting results, in a recent study [8], a total of 119 young male subjects were recruited to perform 50 eccentric contractions consisting of 2 sets of 25 contractions and blood CK activity was analyzed. The groups were divided into high, medium and low responders based on CK activity levels during pre, 24, 48, 72, and 96 h post exercise. The study used an elbow flexor protocol and the results revealed that high CK responders presented a higher body fat percentage (15.56%) when compared with low CK responders (12.83%), while CK activity was not correlated with percent body fat. Another study [30], compared lean and overweight young females submitted to an isokinetic eccentric exercise protocol. Serum CK activity was determined before, immediately after, 24, 48 and 72 h post-exercise. The results of the study demonstrated that overweight (29.5 kg/m^2^;) subjects produced higher and more prolonged alterations on CK activity following eccentric exercise than those with normal body weight. It is interest to note that although obese participants were studied in this study, it would be expected that obese elderly women with a higher percent body fat would present a higher CK response, but this was not the case. Therefore, the effect of body fat on muscle damage analyzed by CK requires further investigation.

Some concern when analyzing studies with responsiveness are required. We certainly agree that our definition of responsiveness was specific to our study population and exercise type (ERE). Limitations in this study such as genetic variation, the use of one exercise as compared with seven exercises in another study [19], absence of a non-obese group and the lack of a specific diet control as the timing and composition of dietary intake can modulate training responses [28]. In addition, we do not know if the exercise stimulus would have been low enough in the small group of LR to induce a CK and NO release. Although, absolute leg extension 10RM was not different between groups and the ERE protocol was rigorously followed for both groups, it is possible that the relative load may have been higher in the HR group, thus causing higher NO and CK response rather than being due to their individual physiology variation. Lastly, the intake of food rich in nitrite/nitrate over 24-48 h prior and in the following two days of blood collection was not controlled.

Furthermore, variables associated with endothelial dysfunction, such as blood pressure, which would strengthen the NO responsiveness as a protective cardiovascular effect (ex. post-exercise hypotension) were not measured. For the sake of clarity, we understand that it is speculative to define a physiological significance of the NO cut-off values at this moment. Furthermore, CK is an indirect and crude measure of muscle damage. Therefore, more data will be required to define the true thresholds for responders for this and other outcomes in acute and chronic delineated studies [31].

Individual responsiveness to training is becoming a very important tool for scientific studies and exercise prescription, avoiding misinterpretation of data and improving individualized exercise prescription. The higher body fat, cholesterol, LDL and lower upper limb fat-free mass in the LR group might represent a deleterious effect of lower serum levels of nitrite. Future studies should be designed to correlate the acute responsiveness to exercise with the chronic adaptation to training.
